# How to build reproducibility into scientific publishing

**DOI:** 10.1038/s44319-026-00909-y

**Published:** 2026-08-20

**Authors:** Csaba Szabo

**Affiliations:** Section of Pharmacology, Department of Oncology, Microbiology and Immunology; Faculty of Science and Medicine, University of Fribourg, Fribourg, Switzerland

**Keywords:** Science Policy & Publishing

## Abstract

The academic publishing system needs to include replication of key experiments wherever possible to meet the replication crisis. A major challenge is to incentive validation and provide funding for it.

**Figure:**
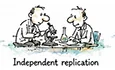

For any biomedical scientific article, the fundamental question is: are its central findings true? In experimental research, the gold standard to address this question is whether independent investigators can obtain the same result when they repeat an experiment. This process is generally termed direct replication, though there are other methods, such as computational reproducibility, where the question is whether independent reanalysis of raw data produces the same conclusion.

The so-called “reproducibility crisis” refers to the widespread problem that independent laboratories, when attempting to repeat a published experiment, fail to obtain the same results. Large preclinical replication efforts in various fields of biomedical science have reported failure rates of 80% or more, due to a combination of factors, including incompletely disclosed reagents or methods, poor design, inadequate controls or statistical power, omitted negative data, and, in some cases, manipulation or fabrication (Szabo, 2025).

Peer review is the standard gatekeeping process in scientific publishing (Fig. 1). Politicians, policymakers, and journalists often use “peer reviewed” as if it meant “independently verified”, “empirically proven”, or simply “true”, without realizing it does not involve independent replication. Reviewers usually do not reanalyze data or examine every image or large dataset in detail; they assess whether the manuscript appears plausible and internally coherent. The question, then, is whether the current scientific assessment/publication process could be reformed with a renewed focus on reproducibility.

**Figure 1:**
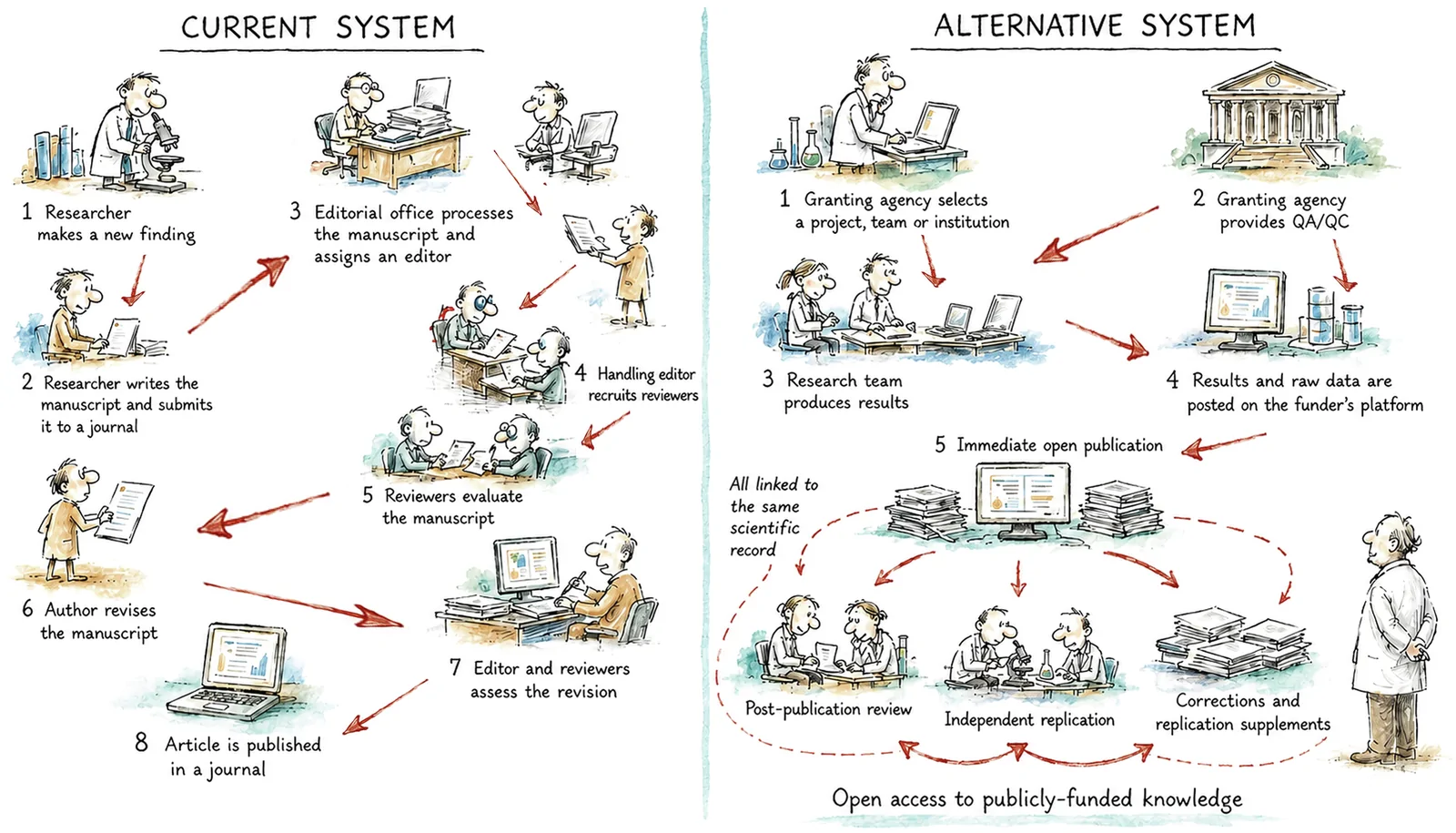
Conventional journal publishing (left) compared to a funder-based publication platform (right). In the current system, a manuscript undergoes editorial processing, reviewer selection, peer review, and revision before it is accepted and published. In the proposed alternative, the funding agency provides the initial quality-control and accountability framework. Results and underlying raw data are posted on the funder’s platform. Scientific evaluation continues after publication through open review, independent replication and corrections.

In many scientific papers, cornerstone experiments are not difficult to identify. A paper may contain many figures and extensive datasets, but usually it is easy to pinpoint those findings that carry most of its conceptual weight. For example, in preclinical research, which often uses standard cell lines, commonly available reagents and routine assays, such cornerstone experiments can often be relatively easily repeated without reproducing the entire study.

A revised publication process should therefore focus less on questions related to ‘novelty’ or ‘disruptiveness’ but pay attention to experimental replicability of key findings. Just like in any other replication work, the endpoints should be specified in advance, appropriate power analysis should be performed, and the experiment should be conducted by an independent group, with appropriate blinding and randomization. The originating laboratory may provide protocols or unusual reagents, but must not control execution or analysis. A failed replication would not by itself prove fraud or invalidate the original conclusion: it could reveal an omitted variable, an inadequately described protocol, or a less robust effect. Each would be important to know before a paper enters the scientific record.

Of course, direct replication would not be feasible for every manuscript. A decade-long cohort study, a unique clinical specimen, or experiments requiring a custom-built instrument cannot simply be repeated during editorial assessment. In such cases, independent reanalysis, validation in an orthogonal model, or later multicenter replication may be appropriate.

One practical model to achieve this goal is the “Replication Supplement” (Szabo, 2025). Authors would arrange for an independent laboratory to repeat the cornerstone experiment before submission. A short report from the replicator group would describe the prespecified replication, methods, and outcome. The main manuscript and this Replication Supplement would be evaluated together, and the report would remain linked to the article, whether the result was positive, negative, or ambiguous. An article accompanied by such a supplement could be guaranteed peer review (not acceptance), which could be a major incentive to conduct the additional work.

Lord et al (2026) have proposed a related, journal-organized model called “Peer Replication”. Authors would opt into the replication track at submission, after which the editor would recruit scientists with the relevant expertise to repeat key experiments. The replication plan would be agreed with the editor and preregistered. Once completed, the results would be published alongside the original paper as a short, citable Peer Replication Report, regardless of whether the replication succeeded or failed. The published main article could then be labeled as “peer replicated”.

One immediate objection is cost. Yes, replication does cost money, but so did every experiment in the original manuscript—as do the “regular” additional experiments routinely requested by peer reviewers. A modest reallocation from novelty production to confirmation could drastically improve the quality of the literature. Funding agencies could also mandate that a portion of research grants should be allocated for independent replication. Making reproducibility relevant to continued support would also quickly change incentives to replicate.

Whichever track is chosen, replication efforts would be most effective if they were combined with a professional scientific assessment process, through paid staff statisticians, methodologists, data forensics specialists, and biomedical scientists (Szabo, 2025; Emanuele and Minoretti, 2025).

Both professional integrity screening and replication work might be, at least in part, financed through an “Integrity Escrow” model. For this model, I propose that manuscript submissions could carry a refundable deposit. A professional in-house integrity unit would screen the submissions for duplication, manipulation, internal inconsistency, statistically implausible patterns, and related warning signs. Honest submitters would receive the deposit back, and the manuscript would proceed to scientific evaluation—ideally with a direct Peer Replication component. However, clear evidence of deliberate fabrication or systematic manipulation would lead to immediate rejection and forfeiture of the escrow. Such a system would have a clear deterrent value. Moreover, its proceeds would be ring-fenced for integrity work and may be used to subsidize the physical replication work of other manuscripts.

Another option to improve the reliability of the scientific literature is to reverse the sequence of events: publish first, then evaluate, verify and, whenever feasible, replicate. *eLife* and platforms such as *Review Commons* or *PeerCommunityIn* already publish selected preprints with public or editorially-organized review reports and editorial assessments. This logic could be taken further: published manuscripts are accompanied by visible “trust markers” signifying integrity screening and statistical assessment (Pinfield et al, 2026).

The main problem I see with the publish-first model is that it is vulnerable to dishonest actors. We already face a tsunami of paper-mill and synthetic submissions, and preprint archives are quickly becoming their latest target. In my view, such a model may work best if the publish-first platforms are under the control of research funders (Fig. 1), such as Open Research Europe, Gates Open Research, or Wellcome Open Research (Szabo, 2026).

Deposit of grant-derived outputs, accompanied by raw data, protocols, analysis code, and essential metadata, should be mandatory and should include negative as well as positive findings. Funding agencies could provide professional quality assessment and quality control, and verify data provenance and data integrity after the work is completed. After publication, scientific evaluation, preferably including replication, would occur openly.

A cautionary precedent is Wellcome Open Research, which has been set up to enable Wellcome-funded researchers to publish rapidly with open data and transparent post-publication peer review. Unfortunately, they only “encourage” and do not mandate funded groups to publish there. A recent analysis found that almost half of the platform’s 2020 output consisted of non-traditional articles that might have encountered difficulties in conventional journals.

None of these platforms has gained a high level of prominence though. As long as journal prestige continues to determine careers and funding, researchers have an obvious incentive to send their papers to established journals. But the lesson here is that funder-based publication can work—if it is made mandatory. For example, if all ERC-funded investigators were required to publish their results in Open Research Europe, the quality of the submitted material would elevate the prominence and attractiveness of this venue.

Ideally, such a scientific publication reform would work only if accompanied by scientific assessment reform (Szabo, 2026) to focus on what matters most. The evaluators should look at what is being published—are the findings robust? Are they replicable? Do they advance the field?—rather than where the publication appears.

## Supplementary information

Peer Review File
