# Supplementary material for: How to build reproducibility into scientific publishing
Source: EMBO Rep. 2026 Aug 20;27(18):5169–71. doi: 10.1038/s44319-026-00909-y (PMC13601543; doi:10.1038/s44319-026-00909-y)
Supplement: Supplementary file 1 [file 44319_2026_909_MOESM1_ESM.pdf]

EMBO reports

Let's build reproducibility into scientific publishing

Csaba Szabó

Corresponding author: Csaba Szabó (csaba.szabo@unifr.ch)

Review Timeline:

Submission Date:	6th Aug 26
Accepted:	14th Aug 26

Editor: Holger Breithaupt

Transaction Report:

This manuscript was editorially reviewed.
